# Supplementary material for: Overland movement in African clawed frogs (Xenopus laevis): a systematic review
Source: PeerJ. 2016 Sep 20;4:e2474. doi: 10.7717/peerj.2474 (PMC5036101; doi:10.7717/peerj.2474)
Supplement: Appendix S1 [file peerj-04-2474-s002.docx]

Supplementary Information

1. Literature used in the systematic review of overland movement in African clawed frogs (*Xenopus laevis*)

Alexander S, and Bellerby C. 1938. Experimental studies on the sexual cycle of the South African clawed toad (*Xenopus laevis*). I. *Journal of Experimental Biology* 15:74-81.

Balinsky BI. 1969. The reproductive ecology of amphibians of the Transvaal Highveld. *Zoologica Africana* 4:37-93.

Channing A. 2001. *Amphibians of central and southern Africa*: Comstock Pub. Associates.

Crayon JJ. 2005. Species account: *Xenopus laevis*. In: Lannoo MJ, ed. *Amphibian Declines: The Conservation Status of United States Species*. Berkeley: University of California Press, 522-525.

Deuchar EM. 1975. *Xenopus: the South African Clawed Frog*. London: John Wiley and Sons.

Du Plessis S. 1966. Stimulation of spawning in *Xenopus laevis* by fowl manure. *Nature* 211:1092.

Eggert C, and Fouquet A. 2005. A preliminary biotelemetric study of a feral invasive *Xenopus laevis* population in France. *Alytes* 23:3-4.

Evans, B.J., Bliss, S.M., Mendel, S.A. & Tinsley, R.C. (2011) The Rift Valley is a major barrier to dispersal of African clawed frogs (*Xenopus*) in Ethiopia. *Molecular Ecology* 20:4216-4230.

Faraone FP, Lillo F, Giacalone G, and Valvo ML. 2008. The large invasive population of *Xenopus laevis* in Sicily, Italy. *Amphibia-Reptilia* 29:405-412.

Fouquet A, and Measey GJ. 2006. Plotting the course of an African clawed frog invasion in Western France. *Animal Biology* 56:95-102.

Herrel A, and Bonneaud C. 2012. Trade-offs between burst performance and maximal exertion capacity in a wild amphibian, *Xenopus tropicalis*. *Journal of Experimental Biology* 215:3106-3111.

Hewitt J, and Power JH. 1913. A list of S. African Lacertilia, Ophidia and Batrachia in the McGregor Museum, Kimberley, with field notes on various species. *Transactions of the Royal Society South Africa* 3:147-176.

Hey D. 1949. A report on the culture of the South African clawed frog *Xenopus laevis* (Daudin) at the Jonkershoek inland fish hatchery. *Transactions of the Royal Society of South Africa* 32:45-54.

Hillman SS, Drewes RC, Hedrick MS, and Withers PC. 2011. Interspecific comparisons of lymph volume and lymphatic fluxes: do lymph reserves and lymph mobilization capacities vary in anurans from different environments? *Physiological and Biochemical Zoology* 84:268-276.

Inger, RF 1968. Amphibia. Exploration du Parc National de la Garamba, Mission H. Saeger, Fascicle 52., pp. 1-190. Institut des Parcs Nationaux du Republique Democratique du Congo, Kinshasa.

Kalk M. 1960. Climate and breeding in *Xenopus laevis*. *South African Journal of Science* 56:271-276.

Kazadi M, de Bruyn L, and Hulselmans J. 1986. Ecological notes on the stomach contents of *Xenopus laevis* (Daudin, 1803)(Amphibia: Anura) collected in Ruanda. *Annales de la Societe Royale Zoologique de Belgique (Belgium)* 116:227-234.

Lillo F, Faraone FP, and Lo Valvo M. 2011. Can the introduction of *Xenopus laevis* affect native amphibian populations? Reduction of reproductive occurrence in presence of the invasive species. *Biological Invasions* 13:1533-1541.

Lobos G, and Garín C. 2002. *Xenopus laevis* (African clawed frog). *Herpetological Review* 33:132.

Lobos G, and Jaksic FM. 2005. The ongoing invasion of African clawed frogs (*Xenopus laevis*) in Chile: causes of concern. *Biodiversity and Conservation* 14:429-439. 10.1007/s10531-004-6403-0

Loveridge A. 1953. Zoological results of a fifth expedition to East Africa. IV Amphibians from Nyasaland and Tete. *Bulletin of the Museum of Comparative Zoology at Harvard University* 110:325-406.

Loveridge J. 1976. Strategies of water conservation in southern African frogs. *Zoologica Africana* 11:319-333.

Mahrdt CR, and Knefler FT. 1972. Pet or pest? The African clawed frog. *Environment Southwest* 446:2-5.

Mahrdt CR, and Knefler FT. 1973. The clawed frog-again. *Environment Southwest* 450:1-3.

Malik AI, and Storey KB. 2009a. Activation of antioxidant defense during dehydration stress in the African clawed frog. *Gene* 442:99-107. 10.1016/j.gene.2009.04.007

Malik AI, and Storey KB. 2009b. Activation of extracellular signal-regulated kinases during dehydration in the African clawed frog, *Xenopus laevis*. *Journal of Experimental Biology* 212:2595-2603.

Mason M, Wang M, and Narins P. 2009. Structure and function of the middle ear apparatus of the aquatic frog, *Xenopus laevis*. *Proceedings of the Institute of Acoustics Institute of Acoustics (Great Britain)* 31:13.

McCoid MJ, and Fritts TH. 1980. Notes on the diet of a feral population of *Xenopus laevis* (Pipidae) in California. *Copeia* 1980:272-275.

Measey GJ. 1997. The ecology of *Xenopus* PhD. Bristol University.

Measey GJ. 1998. Diet of feral *Xenopus laevis* (Daudin) in South Wales, UK. *Journal of Zoology* 246:287-298.

Measey GJ. 2004. Species account: *Xenopus laevis* (Daudin 1802). In: Minter LR, Burger M, Harrison JA, Braack H, Bishop PJ, and Kloepfer D, eds. *Atlas and Red Data Book of the Frogs of South Africa, Lesotho and Swaziland*. Washington DC: Smithsonian Institution Press, 266-267.

Measey GJ, and Tinsley RC. 1998. Feral *Xenopus laevis* in South Wales. *Herpetological Journal* 8:23-27.

Passmore NI, and Carruthers V. 1979. *South African frogs. A complete guide*. Johannesburg: Witwatersrand University Press.

Peralta-García A, Valdez-Villavicencio JH, and Galina-Tessaro P. 2014. African clawed frog (*Xenopus laevis*) in Baja California: a confirmed population and possible ongoing invasion in Mexican watersheds. *The Southwestern Naturalist* 59:431-434.

Picker MD. 1985. Hybridization and habitat selection in *Xenopus gilli* and *Xenopus laevis* in the south-western Cape Province. *Copeia*:574-580.

Poynton JC, and Broadley DG. 1985. Amphibia Zambesiaca 1. Scolecomorphidae, Pipidae, Microhylidae, Hemisidae, Arthroleptidae. *Annals of the Natal Museum* 26:503-553.

Rebelo R, Amaral P, Bernardes M, Oliveira J, Pinheiro P, and Leitao D. 2010. *Xenopus laevis* (Daudin, 1802), a new exotic amphibian in Portugal. *Biological Invasions* 12:3383-3387.

Schramm M. 1987. Control of *Xenopus laevis* (Amphibia: Pipidae) in fish ponds with observations on its threat to fish fry and fingerlings. *Water SA (Pretoria)* 13:53-56.

Simmonds MP. 1985. Interactions between *Xenopus* species in the southwestern Cape Province, South Africa. *South African Journal of Science* 81:200.

Sinsch U. 2006. Orientation and navigation in Amphibia. *Marine and Freshwater Behaviour and Physiology* 39:65-71.

Solís R, Lobos G, Walker SF, Fisher M, and Bosch J. 2010. Presence of *Batrachochytrium dendrobatidis* in feral populations of *Xenopus laevis* in Chile. *Biological Invasions* 12:1641-1646.

Storey KB, and Storey JM. 2012. Aestivation: signaling and hypometabolism. *Journal of Experimental Biology* 215:1425-1433.

Sullivan KB, and Spence KM. 2003. Effects of sublethal concentrations of atrazine and nitrate on metamorphosis of the African clawed frog. *Environmental Toxicology and Chemistry* 22:627-635.

Thurston JP. 1967. The morphology and life-cycle of *Cephalochlamys namaquensis* (Cohn, 1906)(Cestoda: Pseudophyllidea) from *Xenopus muelleri* and *X. laevis*. *Parasitology* 57:187-200.

Tinsley R, and Jackson J. 1998. Correlation of parasite speciation and specificity with host evolutionary relationships. *International journal for parasitology* 28:1573-1582.

Tinsley R, and Sweeting R. 1974. Studies on the biology and taxonomy of *Diplostomulum* (*Tylodelphylus*) *xenopodis* from the African clawed toad, *Xenopus laevis*. *Journal of Helminthology* 48:247-263.

Tinsley R, and Whitear M. 1980. The surface fauna of *Xenopus* skin. *Proceedings of the Royal Society of Edinburgh Section B Biological Sciences* 79:127-130.

Tinsley RC, and Kobel HR. 1996. The Biology of *Xenopus*. Zoological Society of London Series. Oxford: Oxford University Press. p 440.

Tinsley RC, Loumont C, and Kobel HR. 1996. Geographical distribution and ecology. In: Tinsley RC, and Kobel HR, eds. *The Biology of Xenopus*. Oxford: Oxford University Press, 35-59.

Tinsley RC, and McCoid MJ. 1996. Feral populations of *Xenopus* outside Africa. In: Tinsley RC, and Kobel HR, eds. *The Biology of Xenopus*. Oxford: Oxford University Press, 81-94.

Tinsley RC, Stott LC, Viney ME, Mable BK, and Tinsley MC. 2015. Extinction of an introduced warm-climate alien species, *Xenopus laevis*, by extreme weather events. *Biological Invasions* 17:3183-3195.

Vanschoenwinkel B, Gielen S, Vandewaerde H, Seaman M, and Brendonck L. 2008. Relative importance of different dispersal vectors for small aquatic invertebrates in a rock pool metacommunity. *Ecography* 31:567-577.

Wager VA. 1986. *Frogs of South Africa their fascinating life stories*. Johannesburg: Delta Books.

Weisenberger ME. 2011. *Xenopus borealis*: terrestrial activity. *African Herp News* 53:44-45.

Wilson R, James R, and Johnston I. 2000. Thermal acclimation of locomotor performance in tadpoles and adults of the aquatic frog *Xenopus laevis*. *Journal of Comparative Physiology B* 170:117-124.

Wilson RS, James RS, and Van Damme R. 2002. Trade-offs between speed and endurance in the frog *Xenopus laevis* a multi-level approach. *Journal of Experimental Biology* 205:1145-1152.

Yager DD. 1996. Sound production and acoustic communication in *Xenopus borealis*. In: Tinsley RC, and Kobel HR, eds. *The Biology of Xenopus*. Oxford: Oxford University Press, 121-141.

Table S1 Searches made in Web of Science using the scientific name and derivatives of all common names for *Xenopus laevis* (“clawed frog”, “clawed toad” and “platanna”) AND (the Boolean search term to stipulate that the record should contain this AND the next term) four terms related to movement overland (“overland”, “terrestrial”, “migration” and “dispersal”) Numbers refer to the total number of papers resulting from the search, while the number in parentheses refers to those retained for this review. Because searches did not exclude previous results, the total number of unique results retained was only nine.

| Search term | Overland | Terrestrial | Dispersal | Migration |
| --- | --- | --- | --- | --- |
| Clawed Frog | 6(6) | 22(3) | 10 (7) | 22(5) |
| Clawed Toad | 0 | 4(0) | 1(1) | 2(1) |
| Platanna | 0 | 0 | 0 | 0 |
| *Xenopus laevis* | 6(6) | 147(7) | 30 (6) | 738(3) |
